# The effect of preventive administration of metaraminol on hypothermia and shivering in cesarean section patients randomized clinical trial --a randomized controlled study

**DOI:** 10.3389/fphar.2025.1631503

**Published:** 2025-07-07

**Authors:** Letao Yu, Ziyi Zhang, Lili Li, Wenzhe Shen, Qizhu Feng, Chengwen Lei, Jun Shi, Rui Li, Minghong Liu

**Affiliations:** ^1^ Department of Anesthesiology, Second Affiliated Hospital of Anhui Medical University, Hefei, China; ^2^ Department of Anesthesiology, The First Affiliated Hospital of Anhui University of Science and Technology, Huainan, China

**Keywords:** metaraminol, hypothermia, shivering, cesarean section, spinal anesthesia

## Abstract

**Background:**

Hypothermia is a common complication during cesarean section and may lead to a series of adverse outcomes. This study aims to evaluate whether prophylactic low-dose metaraminol infusion, compared to saline control, reduces intraoperative hypothermia and shivering in cesarean section patients under spinal anesthesia.

**Methods:**

This study is a randomized, double-blind, placebo-controlled trial, including a total of 66 full-term pregnant women scheduled for cesarean section under spinal anesthesia. Women were randomly divided into the metaraminol (n = 33) and placebo (n = 33). The metaraminol group was given a prophylactic infusion of metaraminol at a rate of 5 mg/h after the start of anesthesia until the end of surgery, while the placebo group was given an equal volume of saline. The primary outcome was the incidence of hypothermia, while secondary outcomes included the incidence of shivering, changes in rectal temperature, neonatal temperature, and the occurrence of hypotension and bradycardia.

**Result:**

Compared to the placebo group, the metaraminol group significantly reduced the incidence of hypothermia (54.0% vs. 81.0%, P = 0.017) and shivering (27% vs. 57%, P = 0.012). At the end of the surgery, the rectal temperature in the metaraminol group was significantly higher than that in the placebo group (36.72°C ± 0.31°C vs 36.50°C ± 0.26°C, P = 0.003). In addition, the incidence of hypotension was lower in the metaraminol group compared to the placebo group (6% vs. 24%, P = 0.0039).

**Conclusion:**

Prophylactic infusion of low-dose metaraminol during spinal anesthesia for cesarean section can effectively reduce the incidence of hypothermia and shivering. It has a positive impact on maternal temperature and hemodynamic stability, offering a new and effective strategy for temperature management during cesarean section.

## 1 Introduction

During a cesarean section, factors such as anesthesia, environmental temperature, the opening of the abdominal and uterine cavities, the delivery of the fetus and placenta, and intraoperative bleeding can increase maternal heat loss, leading to perioperative hypothermia (Philomena Onyia et al., 2022). The incidence of perioperative hypothermia ranges from 50% to 90% (Zhuo et al., 2022). This contrasts with general anesthesia (30.4% incidence) (Grote et al., 2018), establishing neuraxial techniques as critical targets for thermal management interventions. Intraoperative hypothermia can lead to adverse reactions such as shivering, coagulation dysfunction, wound infection, and delayed healing (Philomena Onyia et al., 2022). This not only affects the mother’s postoperative recovery but may also have adverse effects on the newborn’s health. Therefore, healthcare providers need to closely monitor the mother’s temperature changes, reduce postoperative complications, and ensure the safety of both mother and infant.

Spinal anesthesia is the most commonly used method for cesarean section, praised for its rapid efficacy and minimal impact on both the mother and the newborn. However, spinal anesthesia can cause sympathetic nerve block, which may lead to peripheral vasodilation and increased blood flow, warming the peripheral tissues (Shahin et al., 2021). However, the redistribution of heat due to both non-blocked and blocked areas can lead to a decrease in core temperature. Additionally, spinal anesthesia affects the temperature-regulating center and the lower limb motor nerves, which can lead to shivering in the lower limbs, further decreasing body temperature and resulting in hypothermia (Thakur et al., 2023).

The commonly used drug, metaraminol, as an important neurotransmitter, plays a significant role in physiological regulation within the human body. Due to its ability to act on α-receptors in peripheral vascular smooth muscle, it induces vasoconstriction and increases blood pressure. Literature reports that a prophylactic infusion of 250 μg/min of metaraminol can prevent hypotension after intrathecal anesthesia in cesarean section patients (Philomena Onyia et al., 2022). However, its impact on maternal and neonatal body temperature during surgery remains unknown.

In our pre-test, we infused at different rates of 15 mg/h, 10 mg/h and 5 mg/h and found that 5 mg/h was sufficient to achieve an increase in blood pressure with a low incidence of hypertension among the adverse effects. Therefore, we hypothesised that prophylactic infusion of mesalamine using 5 mg//h would reduce the incidence of hypothermia and chills in patients undergoing cesarean section and positively affect maternal haemodynamics and neonatal temperature.

## 2 Methods

This randomized, double-blind, placebo-controlled trial was conducted from March 2023 to August 2024 at the First Affiliated Hospital of Anhui University of Science and Technology in China. Approved by the Ethics Committee of the First Affiliated Hospital of Anhui University of Science and Technology (2023-KY-B109-001). All participants provided written informed consent. We recruited participants who met the following criteria: ① aged ≥18 years; ② ASA classification I–III; ③ body mass index (BMI) of 27–34 kg/m^2^; ④ planned to undergo emergency cesarean section under spinal anesthesia with a singleton pregnancy. We excluded participants who met any of the following criteria: ① axillary temperature >37.0°C or <35.0°C; ② severe pregnancy-induced hypertension (BP ≥ 180/110 mmHg); ③ contraindications to spinal anesthesia; ④ gestational age ≤34 weeks; ⑤ blood loss ≥600 mL; ⑥ known allergy to the study medication; ⑦ inadequate anesthesia level below T6 or conversion to general anesthesia.

We used block randomization to assign eligible participants into groups. In each block of 10 participants, they were randomly assigned to either group in a 1:1 ratio. The group assignments were concealed within sequentially numbered opaque sealed envelopes. All participants and anesthesiologists were blinded to the group assignments. All patients were randomly divided into the saline group (n = 33) and metaraminol group (n = 33). An independent anesthesia nurse prepared all solutions: Metaraminol group: 1 mL metaraminol (Nanjing Zeheng Pharmaceutical Co.) + 9 mL saline → 10 mL of 0.5 mg/mL solution Control group: 10 mL saline (Wuhan Binhu Shuanghe Pharmaceutical Co., Ltd.) Group assignments used block randomization (10 patients/block, 1:1 ratio) concealed in opaque sequentially numbered envelopes. Patients and anesthesiologists remained blinded throughout the trial. All patients fasted for 8 h and abstained from fluids for 2 h prior to the surgery. Upon entering the operating room, monitoring of electrocardiogram (HR), non-invasive blood pressure (NIBP), blood oxygen saturation (SpO_2_), and forehead temperature was performed, and baseline values were recorded. All parturients were covered with a 3M air-heating warming blanket (upper body) and continuously received low-flow nasal oxygen (3 L/min). An intravenous line was established, and a fluid warmer was used to heat the fluids to 40°C. The patient was positioned in the left lateral position on the operating table, with both hands holding the knees, legs flexed and slightly apart, to fully expose the anal region. Clean the skin around the anus with warm water and sterile gauze to ensure that the area is free of any foreign substances. Use a flexible rectal temperature probe equipped with a GE monitor to measure temperature. Cover with a sterile single-use outer sheath and apply lidocaine cream to both the anal area and the tip of the probe. Gently insert the tip of the temperature probe 5 cm into the patient’s anus and secure it to the inner thigh with tape to measure the core temperature of the patient. The room temperature is set at 22.5°C ± 0.5°C with a humidity of 55%–65%. Ultrasound was used to locate and mark the L3-4 spinous process interspace. The puncture site was disinfected with iodine tincture, covered with sterile drapes, and local anesthesia was administered using 1% lidocaine. The epidural needle was inserted slowly. Upon feeling a distinct loss of resistance, the syringe was aspirated. If no blood or fluid was obtained, it indicated that the epidural space had been reached. The spinal needle was then used to perform a subarachnoid space puncture. After observing the outflow of clear cerebrospinal fluid, 12 mg of ropivacaine hyperbaric 0.5% was injected over a period of 15 s. Subsequently, an epidural catheter was placed 3 cm into the epidural space. The spinal anesthesia procedure was completed within 10 min. At the start of anesthesia, specifically when administering local anesthesia with lidocaine, infusion of the study drug was initiated: 0.5 mg/mL of metaraminol or saline was infused at a rate of 5 mg/h (10 mL/h). The patient was repositioned to a supine position with the operating table tilted 15° to the left. Test the anesthesia level using alcohol swabs. Once the anesthesia level reaches T6, the surgery is initiated.

Monitor vital signs every 5 min from the time the patient enters the operating room. Monitor the maternal rectal temperature at the following time points: upon entering the operating room (T0), after anesthesia (T1), at skin incision (T2), at fetal delivery (T3), at abdominal closure (T4), and at skin suturing (T5). Record the incidence of hypothermia (a temperature decrease of ≥0.5°C). After the delivery of the newborn, collect 2 mL of umbilical artery blood and measure the newborn’s rectal temperature. Record the occurrence of intraoperative hypotension (systolic blood pressure <90 mmHg). In the event of hypotension, administer 6 mg of ephedrine intravenously for treatment. Record the occurrence of hypertension (mean arterial pressure >120% of baseline). The baseline was the mean value of mean arterial pressure measured on admission and on entering to the operating theatre; In case of hypertension, suspend the infusion of the study drug. Resume infusion once the maternal blood pressure returns to below the hypertension threshold. Record the occurrence of bradycardia (heart rate <50 beats per minute). In case of bradycardia, administer 0.3 mg of atropine intravenously for treatment.

During the surgery, observe the occurrence of shivering in the patient and assess it using the Wrench shivering grading scale: Grade 0: No shivering. - Grade 1: Presence of piloerection, peripheral vasoconstriction, or cyanosis, but no muscle tremors. - Grade 2: Muscle tremors occur in one group of muscle. - Grade 3: Muscle tremors occur in more than one group of muscles. - Grade 4: Generalized muscle tremors throughout the body. Calculate the percentage of patients experiencing shivering in each group. Shivering total incidence rate = (Number of grade 1 cases + Number of grade 2 cases + Number of grade 3 cases + Number of grade 4 cases)/Total number of cases × 100%. If the patient experiences grade 3–4 shivering, administer intravenous pethidine 25 mg (Yichang Renfu Pharmaceutical Co., Ltd., batch number: 110,406) and record the treatment.

The primary outcomes observed were rectal temperatures at different time points and the incidence of hypothermia. According to existing literature, the incidence of hypothermia during cesarean section is 80%. Assuming that the incidence of hypothermia in the metaraminol group decreases by 20%, based on α = 0.05 (two-sided) and 1-β = 0.8, with a 1:1 ratio, the required sample size for each group is calculated to be at least 30 participants using the PASS 17.0 software (NCSS, United States). Considering a 10% loss to follow-up rate, the total sample size is set at 66 participants. The secondary outcomes observed include the incidence of shivering, hemodynamic changes, neonatal rectal temperature, fetal umbilical artery blood pH value and adverse reactions.

Statistical analysis shall be conducted using SPSS26.0 statistical software. For continuous variables, data are expressed as mean ± standard deviation ( $\bar{x}\pm s$ ). For non-parametric data, frequencies are reported. The Kolmogorov-Smirnov test is used to assess the normality of the data distribution. If the data follows a normal distribution and the variances are homogeneous, independent sample t-tests are used. If the data does not follow a normal distribution, the Mann-Whitney U test is used for two independent samples. Categorical variables are presented as counts and percentages n (%), and comparisons are made using the chi-square test. A P-value of <0.05 is considered statistically significant. Graphs were created using GraphPad Prism 8 software.

## 3 Results

In our pre-trial involving 40 cesarean section patients randomly assigned to different metaraminol infusion rates (15, 10, and 5 mg/h), the 5 mg/h group demonstrated optimal outcomes: lowest hypertension incidence (2%) with effective hypotension prevention (5.1% incidence). Higher doses showed increased hypertension risk (12% at 10 mg/h; 18% at 15 mg/h) without additional anti-hypotensive benefits. Thus, 5 mg/h was established as the minimal effective dose with optimal safety profile.

We recruited a total of 70 patients for eligibility assessment. Among them, 2 were excluded due to failure to meet inclusion criteria, and 2 had intraoperative blood loss ≥600 mL. Therefore, the final sample size was determined to be 33 patients per group.

There were no statistically significant differences between the two groups in terms of demographic parameters, environmental temperature, surface temperature (forehead temperature), duration of anesthesia procedure, duration of surgery, blood loss, and fluid infusion volume (Table 1).

**TABLE 1:** Basic demographic and clinical characteristics ( $\bar{x}\pm s$ ).

General information	Control group (n = 33)	Metaraminol group (n = 33)	*P value*
Age (years)	31.79 ± 3.01	32.61 ± 5.96	0.483
body mass index (BMI) (kg/m^2^)	30.52 ± 3.06	30.73 ± 2.73	0.769
Height (cm)	164.86 ± 8.4	163.97 ± 9.1	0.681
Environment temperature (°C)	22.67 ± 0.54	22.53 ± 0.42	0.679
Surface temperature (°C)	35.8 ± 0.42	35.9 ± 0.39	0.320
Duration of spinal anesthesia (Min)	8.5 ± 1.5	8.9 ± 1.3	0.251
Duration of surgery (min)	35.89 ± 8.21	36.23 ± 7.47	0.860
Amount of bleeding (mL)	246.19 ± 10.58	247.61 ± 9.57	0.569
Infusion volume (mL)	861.45 ± 123.25	902.72 ± 111.54	0.158

Notes: Compared with the control group, *P < 0.05.

Table 2 lists the rectal temperatures of the two groups of parturients at different time points during the perioperative period. Major results: At the time of fetal delivery (T3), at the end of surgery (T4), and at skin closure (T5), the rectal temperature in metaraminol group was significantly higher than that in the control group, with differences reaching statistical significance (P < 0.05). However, at the time of admission (T0), after anesthesia (T1), and at skin incision (T2), there were no statistically significant differences between the two groups (P > 0.05) (see Table 2).

**TABLE 2:** Comparison of rectal temperatures at different time points between the two groups (°C, ± s) $\bar{x}$ .

Project	Contol group (n = 33)	Metaraminol group (n = 33)	P value
T0	36.93 ± 0.40	37.06 ± 0.38	0.181
T1	36.94 ± 0.39	37.09 ± 0.32	0.092
T2	36.91 ± 0.30	37.05 ± 0.29	0.058
T3	36.75 ± 0.27	36.95 ± 0.33^*^	0.009
T4	36.52 ± 0.29	36.71 ± 0.27^*^	0.007
T5	36.50 ± 0.26	36.72 ± 0.31^*^	0.003

Notes: Compared with the control group, *P < 0.05.

We also compared the differences in rectal temperature changes between consecutive time points from entering the operating room to leaving the operating room. Findings: The difference in temperature change is significantly different at the following points in time (P < 0.05): Difference in rectal temperature change between fetal delivery and skin incision (ΔT3-T2), between closure of the peritoneum and the time of fetal umbilical cord severance (ΔT4-T3), and from entering the operating room to leaving the operating room (ΔT5-T0).

However, Difference in rectal temperature change between the following time points were similar between the two groups and did not show statistically significant differences (P > 0.05): Differences in rectal temperature change between anesthesia and admission (ΔT1-T0), between skin incision and anesthesia (ΔT2-T1), and between skin closure and closure (ΔT5-T4) were similar between the two groups (P > 0.05) (see Table 3).

**TABLE 3:** Comparison of rectal temperature differences at various time points between the two groups (°C, ± s) $\bar{x}$ .

Project	Contol group	Metaraminol group	P value
ΔT1-T0	0.23 ± 0.11	0.25 ± 0.07	0.458
ΔT2-T1	0.31 ± 0.12	0.27 ± 0.14	0.217
ΔT3-T2	0.34 ± 0.13	0.26 ± 0.16^*^	0.029
ΔT4-T3	0.37 ± 0.18	0.29 ± 0.13^*^	0.042
ΔT5-T4	0.36 ± 0.14	0.28 ± 0.19	0.093
ΔT5-T0	0.54 ± 0.27	0.27 ± 0.19^*^	0.0001

Notes: Compared with the control group, *P < 0.05.

Table 4 lists the incidence of hypothermia, shivering, neonatal temperature, fetal umbilical artery blood pH, and adverse reactions for both groups during the perioperative period.

**TABLE 4:** Incidence of hypothermia, shivering, newborn temperature, and adverse reactions in both groups.

Project	Contol group	Metaraminol group	P value
Incidence of hypothermia	27 (81%)	18 (54%)^*^	0.017
Incidence of shivering	19 (57%)	9 (27%)^*^	0.012
Number of uses of pethidine	12 (36%)	5 (15%)^*^	0.048
Hypotension	8 (24%)	2 (6%)^*^	0.039
Hypertension	0	2 (6%)	0.472
Bradycardia	1 (3%)	4 (12%)	0.352
Headache	2 (6%)	4 (12%)	0.668
Vertigo	1 (3%)	3 (9%)	0.606
Newborn temperature	36.59 ± 0.11	36.63 ± 0.07	0.082
Fetal umbilical artery blood pH value	7.30 ± 0.04	7.29 ± 0.05	0.373

Notes: Compared with the control group, *P < 0.05.

In comparing the two groups of parturients with respect to the incidence of hypothermia, shivering, frequency of pethidine use, and hypotension: The incidence in the experimental group was significantly lower than that in the control group (P < 0.05).

There were no statistically significant differences between the two groups in terms of newborn rectal temperature, fetal umbilical artery blood pH value, hypertension, and bradycardia after delivery (P > 0.05).

Table 5 lists the incidence of shivering grades between the two groups. Analysis of shivering severity using the Wrench scale revealed significantly reduced shivering burden in the metaraminol group. Notably, 72.7% of metaraminol-treated patients experienced no shivering (Grade 0) versus 42.4% in controls (P < 0.05).

**TABLE 5:** Incidence of shivering grades between the two groups.

Project	Contol group	Metaraminol group	P value
0 (None)	14 (42.4%)	24 (72.7%)	0.012*
1 (Piloerection)	10 (30.3%)	5 (15.2%)	0.147
2 (Localized tremor)	6 (18.2%)	3 (9.1%)	0.482
3(Generalized tremor)	3 (9.1%)	1 (3.0%)	0.612

Notes: Compared with the control group, *P < 0.05.

## 4 Discussion

The issue of hypothermia during cesarean sections has always been a clinical focus. Chao et al. demonstrated metaraminol’s efficacy in preventing spinal anesthesia-induced hypotension at 250 μg/min, aligning with our low-dose strategy (5 mg/h ≈ 83 μg/min). Our study extends these findings by establishing its novel thermoprotective benefits (Chao et al., 2019). This study found that prophylactic infusion of low-dose metaraminol significantly reduced the incidence of intraoperative hypothermia and shivering in cesarean section patients, which is consistent with previous research on low-dose Noradrenaline (Palanisamy et al., 2022; Hilton et al., 2016). Noradrenaline maintains core temperature effectively by reducing the redistribution of body heat from the core to the periphery after anesthesia through peripheral vasoconstriction (Hu et al., 2022; Theodoraki et al., 2020). Similarly, metaraminol, as another vasopressor (Costa-Pinto et al., 2024), demonstrates similar effects, further confirming the effectiveness of the strategy of preventing temperature drop by constricting blood vessels during cesarean sections.

Metaraminol primarily acts by activating α-receptors on the vascular smooth muscle, leading to vasoconstriction and thereby increasing peripheral vascular resistance (Lina et al., 2022), which reduces heat loss. In addition, it has been found in animal experiments (Martins et al., 2023): for isoflurane and dexmedetomidine-induced hypotension in horses, metaraminol enhances their myocardial contractility, increases cardiac output, and indirectly increases blood flow in the circulation. Together, these effects contribute to the maintenance of core body temperature. In this study, we showed that the rectal temperatures of patients in the metaraminol group were significantly higher than those of the control group after delivery of the fetus and in the later stages of surgery, which may be closely related to the vasoconstrictive effect of metaraminol.

Hypothermia and shivering during cesarean section not only increase maternal discomfort but may also lead to a series of postoperative complications (Zhuo et al., 2022; Tito et al., 2023), such as coagulation dysfunction, wound infection, and delayed wound healing. Therefore, prophylactic administration of metaraminol not only improves the perioperative experience for the mother but may also have positive implications for reducing postoperative complications and promoting recovery. Additionally, metaraminol has demonstrated excellent performance in maintaining hemodynamic stability in mothers, significantly reducing the incidence of hypotension, which is crucial for ensuring surgical safety. Fitzgerald et al. reported metaraminol’s hemodynamic equivalence to norepinephrine with superior bradycardia profile. Our results further demonstrate its multi-target advantages in reducing shivering while maintaining thermal stability (Fitzgerald et al., 2020).

Despite the achievements of this study, several limitations remain. Firstly, Given the peak period of heat loss during surgical exposure, coupled with confounding factors such as mother-infant skin contact, breastfeeding, and ward temperature variations, we did not monitor maternal temperature throughout the entire perioperative period. Secondly, we did not determine the optimal dose of metaraminol required to prevent shivering after spinal anesthesia. Future studies could further explore the effects of different doses of metaraminol on maternal temperature and shivering to determine the optimal dosage. Additionally, individual responses to metaraminol may vary. Future research could further investigate factors influencing the effectiveness of metaraminol, such as maternal age, body constitution, and underlying health conditions, to provide a basis for personalized medication strategies.

## 5 Conclusion

In summary, We confirm that our selected dose aligns with existing evidence: Zhuang (2020) demonstrated that 1.5 μg/kg/min (equivalent to 5 mg/h for 60 kg patients) is optimal for preventing hypotension during cesarean sections, directly supporting our dosage selection. This study indicates that preventive infusion of low-dose metaraminol can significantly reduce the incidence of hypothermia and shivering in cesarean section patients, and has a positive impact on maternal temperature and hemodynamic stability. This finding provides a new strategy for preventing hypothermia during cesarean sections. However, further research is needed to optimize the use of metaraminol and to explore its effects in different individuals.

## Data Availability

The original contributions presented in the study are included in the article/supplementary material, further inquiries can be directed to the corresponding authors.
